# Prodromal dreams

**DOI:** 10.3389/fpsyt.2025.1625811

**Published:** 2025-08-28

**Authors:** Patrick McNamara

**Affiliations:** ^1^ Department of Psychology, National University, San Diego, CA, United States; ^2^ Department of Neurology, Boston University School of Medicine, Boston, MA, United States

**Keywords:** prodromal dreams, REM sleep, threat detection circuit, dream content, interoception

## Abstract

I present a provisional model and promising evidence, pending robust longitudinal validation, for the possibility of prodromal dreams: dreams that predict onset of illness before overt symptoms appear. Interoceptive signals are compressed and summated/integrated within brain networks that are most highly active during REM sleep. If there is a bodily problem an error signal is generated and the brain then attempts to infer a cause or explanation for the distortions in the bodily image. It is this picture or updated model of the body (which attempts to depict or explain the distortions or errors in bodily senses) which I suggest then gets depicted in dreams. Prediction error can be remedied either via active inference leading to corrective action, or by model updating—generating models that explain away the error. I suggest that the depiction of the cause of the predictive error (bodily distortions) emerges in dreams (typically with picture language or metaphor) and thus can be interpreted to help diagnose emerging illnesses. The active inference portion of the model updating process on the other hand will depict potential solutions to the predictive error and if this information emerges in dreams these dreams might plausibly contain information to ameliorate/treat the causes of the bodily distortions.

## Introduction

In medicine, a prodrome can be defined as early sign(s) and/or symptom(s) that may indicate the onset of a disease before more diagnostically specific signs and symptoms emerge. Prodromal dreams are dreams that contain cognitive content (images, scenes, narratives etc.) that significantly predict onset of illness *before any other overt symptoms of that illness manifest*. The questions I seek to answer are: What neural mechanisms cause such dreams? Can they truly be deemed prodromal? Does the content of these dreams truly contain medically valuable information, or information that might assist in either diagnosis or treatment? After answering these questions, I then suggest new directions for research that can build upon what is currently known about prodromal dreams in order to definitively assess their value for psychology and clinical medicine. This essay is not meant to serve as a comprehensive review nor an exhaustive cataloging of all recent work. Rather, I aim to provide the reader with a sampling of representative new research findings on prodromal dreams in hope of ascertaining their potential value in treating mental and physical illnesses. Nor am I systematically assessing the empirical evidence for prodromal dream content. I am merely pointing out that people have for thousands of years reported and recorded such dreams, and I am asking if there are neural mechanisms that could plausibly support the occurrence of such dreams. If there are such mechanisms, then the case for their systematic investigation becomes more plausible.

## Prodromal dreams

As mentioned above prodromal dreams are dreams that contain content that significantly predicts illness *before any other overt symptoms manifest*. Dreams become candidates for prodromal status if the individual (or observer) notices that a change in content of their dreams are the first and earliest manifestation in consciousness of any threat of illness and then that illness emerges. Physicians in the ancient world took dream content seriously and used it to predict illness onset, diagnosis of illness and sometimes to develop prescriptions to treat the illness (see for example reviews in 1) The Russian psychiatrist Vasily Kasatkin collected dreams, including longitudinal series of dreams over a couple of decades from at least 247 patients suffering from a variety of illnesses—some with neurologic/brain damage. While Kasatkin published his findings in a book “The theory of the dream” published in Russian in 1967, as far as I am able to ascertain that book has not been fully translated into English. I obtained a partial translation (2) which reports analysis on dream contents derived from 1642 dreams collected from 247 patients. Only 54 of those dreams reported any feeling of physical pain, but almost all of these dreams, according to Kasatkin contained overt or symbolic/analogical references to or depictions of an impending or existing illness. Dream content furthermore tracked the progression and course of the illness with dream content anticipating features of the illness and afterwards depicting scenes of wellness when recovery occurred. The Kasatkin studies will require further investigation to establish their methodological controls. According to Garfield (3) and van de Castle (4) prodromal dreams have been documented for gastrointestinal, pulmonary, gynecologic and obstetric, dental, and arthritic illness. Shafton (5) reviewed the literature on prodromal dreams available in 1995. He noted that there were an abundance of examples of bodily symptoms appearing in dreams in the psychoanalytical literature. Freud in his *The Interpretation of Dreams* (1900) (in a footnote added in 1911) referred to the work of the German philosopher Karl Albert Scherner whose “Life of the dream” argued that internal somatic stimuli provided the raw material for dream content such that one could deduce the health of particular organs from dream images. A “blazing furnace” could be interpreted as infected lungs in a person coming down with respiratory illness and so on. Shafton (5) provided many examples of dream content heralding illness onset from the extant literature at the time of his review such as persons with dreams of wounds in the left arm or shoulder or jaw or neck predicting later heart attack and so on. For example, Smith (6) found that the number of dream references to death and separation correlated with the severity of cardiac dysfunction, as measured by the ejection fraction, which is a sensitive parameter of disease severity. More recently, Burk (7) reported that warning dreams preceded a breast cancer diagnosis with the women reporting that the dreams they experienced were more vivid, real or intense than ordinary in 83% of reports. A number of quantitative studies of prodromal properties of dreams have tended to suggest that such dreams generally contain higher themes of the dreamer experiencing a variety of threats and aggressions and attempting to respond to them. For example, dreams that precede migraines contain more anger, misfortune, apprehension, and aggressive interactions than do dreams not preceding migraines (8). Building upon several cross-sectional findings of prodromal dreams with high aggressive content predicting onset of Parkinson’s Disease (PD), Otaiku (9) used data obtained from the Parkinson’s Progression Markers Initiative cohort study. Patients were evaluated at baseline and at the 60-month follow-up, with validated clinical scales. Otaiku used the REM Behavior Disorder Questionnaire (RBDSQ) item 2, which inquires whether patients frequently experience aggression in their dreams as the measure of dream content. Results revealed that aggressive dreams predicted a faster increase in motor severity scores (β = 4.64, p = 0.007) and (β = −1.49, p = 0.001).

Furthermore, experiencing consistent aggressive dream content prior to overt onset of PD conferred a 6 fold and 2-fold risk for progressing to more severe motoric PD and more severe cognitive impairment within 60 months.Otaiku (10) later followed up the study on PD patients with a study on healthy middle-aged adults who were followed for many years. Risk of cognitive decline was evaluated in 605 middle-aged adults (mean age = 50 years) from the Midlife in the United States (MIDUS) study who were cognitively normal at baseline and were followed-up for a maximum of 13 years. Cognitive decline was defined as having an annual rate of decline in global cognitive function (measured using five cognitive tests) ≥ 1 standard deviation faster than the mean decline rate from baseline to follow-up. In addition, risk of incident all-cause dementia was evaluated in 2600 older adults (mean age = 83 years) pooled from the Osteoporotic Fractures in Men Study (MrOS) and the Study of Osteoporotic Fractures (SOF), who were dementia-free at baseline, and were followed-up for up a maximum of 7 years. Incident dementia was based on doctor-diagnosis. Frequency of distressing dreams was assessed in all cohorts at baseline (January 2002–March 2012) using an item on dream content of the Pittsburgh Sleep Quality Index questionnaire. Results revealed that compared with middle-aged adults who reported having no distressing dreams at baseline, those who reported having weekly distressing dreams had a 4-fold risk of experiencing cognitive decline and amongst older adults, the difference in dementia risk was 2-fold greater for those reporting distressing dreams. Nightmares and disturbing dreams may also precede onset of the autoimmune disorder (SLE) and flare-ups within SLE (11). In a qualitative interview study Sloan et al. (12) reported that several SLE patients reported that their flare-ups were consistently preceded by disturbing dreams/nightmares which involved being attacked, trapped, crushed, or falling. Prodromal dreams also occur in neuropsychiatric disorders. Basquin et al. (13) found that 43.9% of the 41 patients with bipolar disorder (BPD) they surveyed had experienced prodromal dreaming changes a median of 5 months before the onset of a manic or depressive episode. These dream content changes included nightmares (followed by awakening), bad dreams (not followed by awakening), epic dreaming (feeling of excessive dreaming), dreams involving suicidal scenarios, sleep talking, sleepwalking, and hypnagogic (while falling asleep) and hypnopompic (while awakening) hallucinations. Geoffrey et al (14) investigated the sequence and evolution of dream content changes before a suicidal crisis in depressed patients admitted to a psychiatric emergency department after a suicide attempt or due to suicidal thoughts with immediate intent. Three different alterations were identified: bad dreams (dysphoric dreams without awakening), nightmares (dysphoric dreams with awakening), and suicidal scenarios in dreams. The study found that 80% of individuals had altered dreams in the months leading up to a suicidal crisis. Furthermore, a timeline and progression of these dream content changes were noted: bad dreams began appearing 4 months before a crisis, followed by nightmares 3 months prior to the crisis, and suicidal scenarios in dreams 1.5 months before an attempt. Prodromal dreams are also common when an infection is imminent. Šćepanović et al. (15) utilized a deep-learning algorithm that extracted mentions of medical conditions from text and applied it to two datasets collected during the recent COVID pandemic: 2,888 dream reports (dreaming life experiences) and 57 million tweets (waking life experiences) mentioning the pandemic. Analyses of these datasets revealed that health expressions common to both sets were phrases concerned with or depicting typical COVID-19 symptoms (e.g., cough, fever, and anxiety). Expressions in waking life, however, described symptoms in realistic or common-sensical ways (e.g., nasal pain, SARS, H1N1), while those from dreams appeared to be more metaphorical and uncommon (e.g., maggots, deformities, snake bites) or conditions of surreal nature (e.g., teeth falling out, body crumbling into sand). The dream-related accounts of the COVID experience often appear before onset of COVID diagnosis and then continue during the illness period. The dream content appears to be exploring the outer edges or difficult-to-define margins of the experience. In short, dreams both predicted and captured/described a larger part of the COVID experience than discursive waking reflections on the experience.

The above summary of recent representative studies of prodromal dreams provides a prima facie case that such dreams may exist, though it certainly does not establish that they in fact exist. My purpose here was simply to highlight that existing evidence suggests some potentially interesting content in dreams that may be related to a variety of bodily illnesses. That content, furthermore, in some cases significantly predicts illness before overt illness emerges, and so may be non-trivially related to the course of the illness itself. There is also no single type of content that appears to be prodromal in nature. No single dream image or item seems to consistently signal impending illness, though of course these preliminary conclusions are just that—preliminary. My conclusion is that this cursory look at representative studies suggests that such dreams may repay further investigation. If potential neural mechanisms to support such associations between dream content and impending illness exist then the study of such dreams might be made more rational.

I begin my examination of these dreams by first asking what kinds of brain mechanisms may produce such dreams as even provisional identification of the mechanisms involved may help to clarify how such dreams might provide useful information on health and illness.

## Sleep, dreams and health

The average adult human enters sleep through non-REM (NREM or N1, N2 and N3) sleep phase N1 and then descends into deep slow wave sleep (SWS). There are 3 NREM phases N1, N2, and N3 or slow wave sleep (SWS). After about an hour of NREM sleep, we leave deep SWS as sleep lightens and we briefly pass back up through stages N2 and N1; and the electroencephalographic or EEG wave form gets faster and faster, almost resembling waking activity. The slow, rolling eye movements, characteristic of N3, begin to show periodic spikes with the eyes darting back and forth under the closed eyelids (rapid eye movements or REM). Muscle tone is lost and we are unable to move. These dramatic changes signal the onset of the first episode of REM sleep of the night. Once we enter this first REM episode we will have undergone one full NREM to REM cycle. While this first REM period will last about only ten to twenty minutes, by the time morning comes these REM episodes will last about thirty to forty minutes. The last long REM period which occurs towards morning is the period when most dreams are recalled.

Most recalled dreams tend to be highly visual and emotional narratives and likely come from REM sleep (16). REM sleep is a state characterized by occurrence of rapid eye movements (REM), electroencephalographic activation comparable to waking consciousness, muscle atonia, hippocampal theta oscillations, and ponto-geniculo-occipital (PGO) waves, which are field potentials that are similar to “orienting reactions” generated after a challenge/threat/surprise. They are generated from the cholinergic brain stem and pons, lateral geniculate nucleus, and striate/extrastriate cortex, and vivid dreaming. 5HT2A (5-hydroxytryptamine) receptors and GABA-ergic (γ-aminobutyric acid-ergic) neurons localized in the ventrolateral periaqueductal grey (vlPAG) function to modulate REM-on neurons. In addition, tract tracing studies in monkeys have demonstrated that VENs (Von Economo Neurons) project to the PAG and parabrachial nucleus (PBN), indicating a role for von Economo neurons or VENs in regulation of PAG-related and sleep related functions (17). Thus, PAG and likely 5HT2A receptors are key neural systems implicated in REM onset, maintenance and intensity (18, 19). During REM dreaming external sensory input is reduced, cholinergic and dopaminergic activity levels are high, while serotoninergic and noradrenergic levels are low or absent, thus enhancing brain plasticity and pushing the brain-mind into a visually and emotionally driven, hyper-associative state (20–23). fMRI and rCBF scanning with simultaneous EEG scalp recordings of healthy participants asleep in the scanner demonstrate selective activation of the pontine tegmentum, thalamic nuclei, several limbic elements including the amygdala and the hippocampus, anterior cingulate cortex, insula, mediobasal prefrontal lobes, temporal-parietal-occipital (TPO) junction and extrastriate cortex (24–26). In contrast, the dorsolateral prefrontal cortex is downregulated in REM sleep (16, 27, 28 In non-REM phases of sleep there is a functional uncoupling of the default mode network or DMN’s anterior and posterior nodes (particularly the MPFC and PCC) which are then re-coupled with the onset of REM (29). In summary, REM appears to promote intense visual simulations of socio-emotional associative processing largely in the absence of reflective thought.

Prodromal dreams would likely not be possible if sleep states were not significantly involved in maintaining health of the individual. Sleep states likely maintain health and promote recovery from illness when illness occurs. The sleep-related contribution to health may extend to dreams. If prodromal dreams occur then an additional way in which sleep states facilitate health is to help prepare the individual for an impending illness. I will therefore now summarize just a few salient facts concerning the influence of sleep states on key health parameters. Two major sleep states (N3 and REM) appear to be homeostatically regulated. By homeostatically regulated we mean that the amount and intensity of sleep an individual experiences is controlled by a kind of internal thermostat. If you get too little sleep you cumulate a sleep debt that needs to eventually be paid back or made up. To make up for lost sleep time you sleep a bit longer and a bit more intensely on subsequent nights. In short, you sleep in proportion to wake time. The longer the wake time (or the greater the amount of sleep deprivation), the greater the subsequent sleep time and intensity. Homeostatic regulation indicates that sleep is a physical need and very likely fundamental to health or else it would not be an obligate physiologic process. We do not know if dreams themselves are homeostatically regulated though there are some facts consistent with such a claim. Individuals who are on medications that suppress REM sleep for long periods of time tend to report less dreaming during that time and then abundant dreaming (suggesting dream rebound) when the medications are discontinued. For example, in his review of medications that have significant secondary inhibitory effects on REM, Naiman (30) identifies the benzodiazepines, the anti-depressants (particularly the tricyclics and the older monoamine oxidase inhibitors) and the anticholinergics as major culprits in an epidemic of REM/dream loss. The anti-cholinergic in particular strongly suppress the activity of acetylcholine, the primary neurotransmitter responsible for initiating REM. These anti-cholinergic medications are widely used to treat a very wide variety of illnesses from gastrointestinal disorders to everyday allergies. The likelihood of using anticholinergic drugs increases with age as does the *anticholinergic burden*—a measure of the serious cumulative side effects associated with increased use. Discontinuing use of any of these anti-cholinergics is always accompanied by reports of increased and vivid dreaming.

Similarly, when we experience sleep deprivation over a period of a few days, we normally experience, not only sleep rebound but very vivid and abundant dreams (dream rebound) when we are allowed to sleep again. Sleep also plays a crucial role in the regulation of the immune system, acting as a fundamental process for maintaining immune homeostasis and enhancing the body’s defense mechanisms (31). During slow-wave sleep (SWS) the production and release of cytokines such as interleukin-1 (IL-1) and tumor necrosis factor (TNF), increase. Sleep also facilitates the proliferation of T cells, a type of white blood cell that is critical for identifying and eliminating pathogens. Sleep, furthermore, influences the balance between pro-inflammatory and anti-inflammatory responses, which is vital for preventing chronic inflammation and autoimmune conditions. Sleep deprivation, for example, decreases antioxidant levels thus leading to oxidative stress. Both NREM SWS and REM are associated with changes in levels of circulating neurohormones such as growth hormone, oxytocin, vasopressin and the glucocorticoids with all kinds of consequences for mood regulation and related health issues (32). Both REM and NREM sleep states also bidirectionally interact with the gut microbiome particularly affecting the delicate balance of the gut microbiota (33). Sleep is known to be essential to the glymphatic clearance system. Because the brain is a highly metabolically active organ it generates substantial waste products that require efficient clearance mechanisms. It does so via the glymphatic system among other systems. The glymphatic system relies on the active participation of glial cells, particularly astrocytes, to facilitate the efficient removal of waste products and maintain the brain’s homeostasis. A crucial component of this system is the water channel protein Aquaporin-4 (AQP4), predominantly expressed on astrocytic endfeet surrounding blood vessels. These endfeet create a structural framework enabling the convective flow of cerebrospinal fluid (CSF) into the brain parenchyma, allowing for the exchange of water and solutes between the CSF and interstitial fluid. The glymphatic clearance system is activated during NREM and may even depend on the rolling waves associated with slow wave sleep. The expansion of the interstitial space during sleep enhances the convective flow of CSF, facilitating the efficient removal of waste products.

Now that we have established that sleep and dreams appear to be required for health, we next inquire into *how* prodromal dreams might occur. To see that we first need to lay out the case for the fact that REM sleep neurobiology (the neurobiology most strongly associated with vivid detailed dreams) is associated with a brain network or circuit that specializes in threat detection—both external threat detection and internal (interoception) threat detection. After all, if prodromal dreams constitute early warning signals that illness is imminent, then presumably they are communicating/displaying/depicting information picked-up by an internal threat detection system sensitive to interoceptive signaling systems.

## REM sleep and the threat detection and response systems

According to Wen et al. (34) consensus among experts studying this issue is that there is a threat circuit that is composed of the amygdala, periaqueductal gray, hippocampus, medial prefrontal cortex, and insular cortex. This consensus is consistent with the early proposal for a threat detection circuit by Whalen (35) who proposed a threat activated vigilance system centered on the extended amygdala and related structures such as the nucleus basalis of Meynert. But Wen et al. also present new data which suggests an extended threat detection circuit that includes these amygdala related networks but also involving higher order cognitive systems as well. Consistent with Wen et al., some investigators (36, 37) have argued that an ‘innate alarm system’ (IAS), is constituted by a network of interconnected brain regions including the brainstem, amygdala, pulvinar, but also the higher-order frontal-temporal cortex. Like other threat detection schemes, this IAS is centered on the amygdala and while the amygdala communicates threat information to the prefrontal cortex, it also communicates with subcortical sites such as the locus coeruleus (LC), and the periaqueductal gray (PAG), where both of these structures work together to elicit ‘fight-or-flight’ responses to threat. Interestingly, the cerebello-limbic-thalamo-cortical network, is also suggested to play a role in the ‘innate alarm system’ where it is in constant communication with midbrain structures and is thought important for processing rapid responses to threat. Barrett and Simmons (38) proposed an interoceptive threat detection system consisting of paralimbic sites like the amygdala, medial and anterior cingulate cortex, the posterior ventromedial prefrontal cortex, the posterior orbitofrontal cortex, and the anterior insular cortex (AIC). This interoceptive threat detection system transmits information through connections in the amygdala, hypothalamus, ventral striatum, and periaqueductal gray to the spinal cord that detects autonomic, hormonal, and immunologic (etc.) problems.

Clearly, threat detection brain processes tend to center on the operations of the amygdala and related paralimbic networks. The amygdala is known to process aversive information from multiple internal and external sensory modalities. Lesioning the amygdala attenuates normal responses to dangerous stimuli, and humans with bilateral damage to the amygdala exhibit reduced or no fear responses to most external threats. The amygdala is densely interconnected with the hippocampus, insula (including the anterior insular cortex or AIC), and medial prefrontal cortex or PFC and the temporal parietal occipital junction (TPO)—all sites important in REM sleep. Indeed, TPO lesions are associated with both loss of dream recall and intense bodily distortions (39, 40). In summary, the neural networks most often proposed to function as threat detection circuits are differentially activated during REM relative to waking state activation patterns.

## Brain sites activated during REM sleep

Although dreaming occurs throughout sleep awakenings from REM sleep show greater frequency of dream recall and result in longer, more vivid dream reports (16). Neuroimaging studies have identified a distinct midline anterior paralimbic area that becomes selectively activated during REM sleep, often to levels similar to or exceeding waking (25, 27, 28, 41–45). Solms (39) and Doricchi and Violani (40) report that parietal lobe involvement differentiates patients with and without global cessation of dreaming (GCD) with 42% of GCD patients exhibiting parietal lesions, in particular near or in the region of the temporal parietal occipital junction (TPO) area. At the neuroanatomical level, the temporoparietal junction plays a critical role in interoception and multisensory integration, thus mediating the sense of a bodily image/self (39, 46, 47).

If REM sleep brain activation patterns overlap or clearly involve some of the same networks as the proposed structures frequently cited as threat detection networks, then it may be that REM processing systems could be involved in internal threat detection as well as interoception more broadly. We therefore need to next discuss interoception and its relation to sleep and dreams.

## Interoception

Interoception refers to a set of processes by which internal bodily stimuli (and perhaps skin/touch) are detected, interpreted, and integrated by the brain into an overall picture of the of the body (48). Garfinkel et al. (49) suggested 3 measures of interoception: interoceptive accuracy, sensibility, and overall awareness. These measures can evidence abnormality in mental and physical disorders. If abnormal values on these measures persist over time one may develop the enduring sense that something is off, and one’s sense of self may be associated with chronic distress and anxiety. Indeed, distorted interoceptive signals may lead to disturbances in the sense of body ownership or agency (50). Over time, the constant sense of uncertainty around the cause of bodily sensations may lead to a generalized sense of anxiety and distress as well as might be called a shaky sense of self. Interoceptive disturbances will also lead to REM sleep and dream disturbances as well.

## REM sleep and interoception

In the normal situation with onset of REM sleep, there is a generalized reduction of homeostatic regulation of autonomic reflexes (respiratory, cardiovascular, thermal) during REM (51). *This suspension of normal interoceptive functioning suggests that interoceptive integration and updating processes take place during REM*. Ongoing interoception is briefly suspended so that interoceptive integration can proceed unimpeded. Note that ongoing interoception is different from interoceptive integration. I emphasize that what I am arguing is that interoceptive integration rather than ongoing interoceptive signaling takes place in REM and this is what makes prodromal content possible. The existing neurobiological evidence summarized below shows that multidimensional sensory information is compressed into highly processed unidimensional information in the structures (such as the amygdala and the insula) that are highly activated during REM. Dreams appear to reflect the fact that non-compressed sensory information is not normally processed in dreams given that the sensory composition of dreams generally lacks signals from interoceptive systems and is strongly weighted toward hallucination of the exteroceptive visual sense (16, 52 *). Instead what IS processed in REM-related dreams is information that is highly compressed into summative information about the integrity of bodily systems.* The relatively common experience of movement and conversation/thinking in dreams suggests that fictive proprioceptive and sensorimotor sensations are also operative and generated (53). The reason dreams do not represent primary interoceptive stimuli very frequently, at least in the healthy state, is likely due to the fact that interoception integration involves considerable data compression during REM. Multidimensional physiologic signals become progressively more unidimensional as they undergo compression and integration into cortical synthesis networks (amygdala, AIC, vmPFC and TPO) that are activated during REM (on interoceptive data compression see 54). In compression, some sense data are retained, whereas other sense data are permanently discarded. In integration, sense data from different pathways and even modalities are combined to form lower-dimensional summary representations. Interestingly brain areas which handle compression and integration tend to be agranular (without layer IV thalamic relay) (38). These are precisely the limbic and paralimbic regions activated during REM. These include anterior temporal lobe, anterior cingulate cortex, ventromedial prefrontal cortex, ventral anterior insula, and posterior medial orbitofrontal cortex. Many of these regions are also known as ‘heteromodal association regions’, and are capable of representing low-dimensional (i.e., compressed) and multimodal (i.e., integrated) representations that are abstracted from primary high-dimensional sense data.

Now once interoceptive signals are compressed and summated/integrated the organism gets a snapshot picture of the overall health of the body. If there is a problem an error signal is generated and the brain then attempts to infer a cause or explanation for the distortions in the bodily image. *It is this picture or updated model of the body (which attempts to depict or explain the distortions or errors in bodily senses) which I suggest then gets depicted in dreams.* Note however that I am not suggesting that a particular dream content is exclusively or primarily derived from this bodily signal. It is unlikely that any single dream content or dream image can be tied to any single bodily signal. Instead, dream content is very likely over-determined by multiple sources. Nevertheless, the fact that interoceptive signaling is plausibly registered in REM dreams suggests that this information might be recoverable, at least to some extent, from dream content. In short, it is possible that the dream, at least in part, portrays the read-out, so to speak, of the explanation for the error signals coming from interoceptive senses.

Within the predictive processing framework (PPF) that read-out is known as predictive error. The brain aims to minimize the discrepancy between the model-based predicted and actual sensory input (*prediction error*). If prediction error is small, there is no need to revise the model, but if the prediction error is large enough, then the model fails to accurately represent the causes of sensory experience and should be updated in order to depict or explain the causes of the bodily distortions (55). Prediction error can be remedied either via active inference leading to corrective action, or by model updating—generating models that explain away the error. I suggest that the depiction of the cause of the predictive error (bodily distortions) emerges in dreams (typically with picture language or metaphor) and thus can be interpreted to help diagnose emerging illnesses. The active inference portion of the model updating process on the other hand will depict potential solutions to the predictive error and if this information emerges in dreams these dreams might plausibly contain information to ameliorate/treat the causes of the bodily distortions. However, it should be noted here that none of these reflections should be taken as recommendations that an individual should look to their dreams for potential medical treatments. My aim here is merely to point to the possibility that some dreams may contain content that depicts potential “treatments” for impending illness. This would have been adaptive in the ancestral state. For example, if ancestral humans faced an infectious disease outbreak, persons who had dreams that nudged them into a pattern of social distancing (for example experiencing other people as hostile and threatening) would have been more likely to survive that outbreak than individuals who had no such dreams. In today’s world if individuals began to recall dream content that similarly nudged them into distancing themselves socially from others, it might conceivably portend some sort of infectious outbreak. Of course, this suggestion is entirely speculative and would need to be tested rigorously with large-scale dream content and longitudinal associations of that content with illness patterns before it could be taken seriously. Nevertheless, it seems worth considering given the sleep and dream physiologies we have reviewed here.

The extent to which brain areas are granulated and laminated seem to be of particular importance for active inference. More granular and more highly laminated brain tissue likely support largely unimodal, high-dimensional representations that unfold with a quicker rate of change. A good example of brain regions activated during REM and that exemplify these cellular computational and functional patterns according to Feldman et al. (54) is the insula (see also 56 for a review of insula functions). The human insula contains a dorsal posterior-to-ventral anterior laminar gradient, wherein predictions (depicting solutions to errors) emanate from ventral anterior regions and prediction error (depicting state of the organism) from dorsal posterior regions. Interoceptive sense data is thus thought to become increasingly compressed and integrated as it moves from posterior to mid- to anterior insula. Within the insula itself, interoceptive sense data is progressively compressed and integrated into lower-dimensional representations as information propagates from the dorsal posterior to the ventral anterior of the structure The insula transitions from granular cortical tissue with four layers in its most posterior extent, to dysgranular cortex (with a rudimentary layer IV) in its middle extent, to agranular cortical tissue with three layers in its most ventral and anterior extent. According to the Embodied Predictive Interoceptive Coding (EPIC) model (38, 54), dorsal and posterior insula receive predictions from agranular and dysgranular visceromotor control regions of the brain, including ventral anterior insula, mid-cingulate cortex and anterior cingulate cortex, posterior ventromedial prefrontal cortex, and orbitofrontal cortex. Interestingly, the anterior insula is distinguished by a unique cellular component, the von Economo neurons (VENs) 57). VENs are characterized by large spindle-shaped cell bodies with a single basal dendrite. They are high conduction velocity, long range projection and specialized neurons that are often called the “empathy cells” because their degeneration is associated with diminished empathy for others, self-awareness, social cognition, moral reasoning, and emotional intelligence (58). Within the context of prodromal dreams I suggest these brain regions with the insula at its hub are generating active inference predictive models which respond to prediction errors and thus potentially contain information on how to correct bodily problems in case of illness.

## Dream content and dream theories relevant to prodromal dreams

Take for example, the dreams associated with episodes of sleep paralysis. Sleep paralysis (SP) is a transient experience of conscious paralysis when transitioning into sleep or out of sleep (59). Normally REM sleep is associated with atonia or paralysis that lasts the entire REM episode but occasionally the paralysis persists into the waking state so the person is conscious but cannot move. A recent meta-analysis (60) involving 76 studies from 25 countries with 167,133 participants estimated the global prevalence of SP at 30%. Subjective phenomena associated with SP involve dreams, hallucinations or perceptions of “intruders” that are in the room of the dreamer and intend the dreamer harm. Occasionally a “succubi” is dreamed as sitting on the chest of the victim and assaulting the victim. Presumably the mind is trying to explain why the body suddenly cannot move. Its REM-associated threat detection system is activated given that the brain remains in REM. One possible neural explanation of what is occurring is that a prediction error is generated ultimately in the area of the posterior insula resulting in depiction of the sleep paralysis problem as “there is an intruder in the room and then on the chest and that intends the dreamer harm”. Model updating ultimately emerges in the anterior insula which sends sensorimotor information to move, shout, fully wake up etc. Campillo-Ferrer et al. (61) provide a fuller explanatory account of sleep paralysis and related phenomena taking into account roles of the insula and the TPO. But the basic trigger for model updating processes which produce dramatic changes in dream content and then waking consciousness itself is the threat detection system with REM and embodied in the brain networks that are activated during REM including the insula and its connections with the TPO and amygdala-hippocampal networks.

### Threat simulation theory

Indeed many dreams contain simulations of threats (62). That is not surprising if REM neurobiology involves activation of a threat detection circuit. The threat simulation theory of dreams and the social simulation theory (62, 63) posit that dreams are biased to simulate threatening and social situations. This threat simulation mechanism is thought to consist of two parts: (1) threat recognition simulation which serves to recognize threats faster over time and (2) threat avoidance simulation which serves to implicitly rehearse the appropriate response to that threat (62). In the scheme presented in this paper the first part involves computations of prediction error and the second part involves active inference model updating. While some studies provide support for threat simulation theory or TST (e.g., 64, 65), others do not (e.g., 66). It seems clear however that at least one of the functions of dreams involve threat detection/simulation and generation of simulations on how to avoid the threat in the future. If that is correct and if some of the threat simulations involve interceptive signaling then some dreams should contain information that is valuable for diagnosis of emerging illnesses.

### Sentinel theory

Another theory concerning REM sleep and dreams that is relevant for prodromal dreams is so-called sentinel theory (67). The basic idea is that REM evolved to counteract the deep quiescence of the organism associated with NREM slow wave sleep as that deep sleep made the organism vulnerable to predators. Snyder proposed that periodic awakenings associated with shifts between NREM and REM sleep served the purpose of detecting threats if any existed. Sentinel-like behaviour could also be achieved through variation in sleep timing, periodic awakenings, and periods of time spent in lighter stages of sleep, from which arousals would be more likely in the context of external threatening stimuli. Vertes (68) later developed the sentinel theory further adding the point that the highly activated nature of the brain during REM sleep and dreams may indeed function to counteract the unresponsiveness of slow wave non-REM sleep. Indeed the brain is not completely shut off from the environment during REM sleep but continues to process external stimuli (e.g. 69).

In short, we have initial theoretical frameworks, threat simulation theory and sentinel theory that are consistent with the idea that some REM sleep dreams could act as windows or tools in the detection of both interoceptive and exteroceptive threats and if that is the case then they may contain valuable diagnostic information on bodily integrity and health.

## Methodological issues and recommendations for future research

I have argued that prodromal dreams are real in the sense that changes in dream content significantly predict emergence of an illness typically soon after those changes in dream content and sometimes years before significant illness occurs. I have also discussed how prodromal dreams are consistent with current theory of the threat simulation functions of both REM sleep brain activation patterns and of dream content. I have discussed links between interoceptive signals and their registration in dream content. I believe that these facts provide an initial rationale that justifies further investigation into prodromal dreams. It seems possible that such dreams can provide non-trivial information into detection of emerging illnesses, their properties and even, possibly ways to treat them. But is the effort to capture such information from dreams worth it? And just as importantly is the effort methodologically feasible?

In the last section of the paper I will attempt to provide initial answers to these questions and suggest directions for future research.

### Methodological issues

A potential source of systematic error in the study of prodromal dreams lies in the *post hoc* selection of dream images that appear to match the illness (selection bias). To guard against arbitrary identification of content as prodromal, future empirical studies must adopt both blinded manual coding procedures, and automated identification of potential prodromal content along with confidence ratings on that content as well as pre-registered study designs and robust statistical controls to ensure reliable predictive findings.

### Develop baseline norms about dream content in association with various illnesses and develop a reliable indicator or code regarding how the dream represents threat

To estimate the clinical seriousness of a bodily danger signal we need to know how far that signal deviates from norms. We therefore need to develop dream content norms around associations between various content indices and various types of illnesses. When applying baseline dream-content norms, these also need to be statistical adjustments for individual factors. For example, a firefighter may naturally dream more frequently about burning buildings (without having a lung disease) than the average dreamer. In addition, we would need to take into account differing types and courses of illness. An impending infection is a very different type of illness from mood dysfunction and suicidal ideation. The predictive processing framework distinguishes a hierarchy of predictive model systems with cognitive systems near the top and interoceptive models lower in the hierarchy. Therefore in principle errors signals emanating from interoceptive vs cognitive models may be distinguishable in dream content and thus there may be a signal in dream content that distinguishes these differing types of illnesses. In addition, chronic illnesses that have a course over several years would be expected to be handled differently than acute illness.

If we had reliable information on how the dream codes bodily threat in particular then we could flag such dreams as red flags potentially indicating trouble ahead. But that code would have to take into account baseline frequencies of the threat code. One candidate for such a threat code I suggest is the presence of strangers in dreams and their inferred intentions toward the dreamer. In a study of 320 dream reports from 33 adults, Kahn et al. (70) reported that 48 percent of characters in dreams were known to the dreamer. The average report length was 237 words and contained an average of 3.7 characters. According to the Hall/Van de Castle norms, only about half of the characters in dreams are familiar to the dreamer. In the background of most REM dreams there lurks unidentified characters, people that the dreamer cannot identify. Nevertheless, the dreamer could often tell that the strangers or unidentified people in the dream were vaguely threatening and usually males. In some dream series, up to 80 percent of characters are unknown males who are threatening to the dreamer. Strauch and Meir (71) reported that in about every third dream the dreamer encountered only strangers! In an early study of more than 1,000 thousand dreams, Hall (72) reported (1) that strangers in dreams were most often male; (2) that aggressive encounters were more likely to occur in interactions with an unknown male than with an unknown female or a familiar male or female; and (3) that unknown males appeared more frequently in dreams of males than of females. Domhoff (73) has shown that when male strangers appear in the dream, the likelihood that physical aggression against the dreamer will occur in that dream far exceeds what would be expected on the basis of chance. I suggest that when the percent of unknown characters in a dream exceed baseline frequency (which according to the Hall van de Castle norms is about 50%) then the dream becomes a candidate for prodromal status.

In the effort to develop validated scoring methods or potential prodromal content we should use objective methods like AI, machine learning and classification algorithms, natural language processing and related methodologies to add an objective, automatable, and reproducible solution for a coding system for prodromal dreams.

Another easy to implement strategy to assess the prospects for prodromal dreams is to update the Dream Threat Scale (DTS; 74) to insure that it captures interoceptive signals and then use it in studies that attempt to associate dream content with illness symptomology. The DTS is a content analysis method developed for identifying and categorizing threatening events in dream reports. It captures the Nature of the Threatening Event, the Target of the Threat, the Severity of the Threatening Event for the Self, the Possibility of Actively Participating and Reacting to the Threat, the Participation of the Dream Self in the Event, the Nature of the Reaction of the Self to the Threatening Event, the Resolution of the Threat, and the Realistic Nature of the Threatening Event. But unfortunately it does not currently specifically query interoceptive stimuli. There are now several self-report instruments (75) that specifically assess the multidimensional nature of interoceptive stimuli such as the Multidimensional Assessment of Interoceptive Awareness (MAIA; 76, 77).

### Conduct longitudinal and other controlled studies on the relation of dream content changes to emerging illnesses

Retrospective dream studies, which entail the collection of material only after the onset of the disease, are susceptible to confirmation bias. Therefore longitudinal studies will be necessary to quantitatively assess strength of association and causal directions of changes in dream content to changes in physical and mental health. In addition, traditional case–control type studies should occur such as using a control group design where a sample of individuals without a diagnosed condition recall and provide dream reports, and these are compared to a target group who end-up having an illness. The control group without illness should not be dreaming content comparable to the target group.

### Look at prodromal content in Ultra High Risk populations

A reviewer of an earlier version of this paper suggested: A potential area for future research could be the examination of Ultra High Risk (UHR) populations for psychiatric disorders, with a particular focus on psychosis. These individuals, who have already been identified using validated clinical criteria to be at high risk for a future illness, represent an ideal target population for mid-term longitudinal studies. This is since they have a high risk of conversion and can be followed in an ethical and controlled manner. Prodromal content could be related to conversion rates and odds ratios for conversion to psychotic episodes. Some of the dream content that would be worth assessing for prediction of psychotic onset would be bizarre elements in dreams, aggressive strangers in dreams, perceptual distortions, and general threat level in dreams (perhaps assessed with DTS or sentiment analyses using natural language processing and machine learning tools). that occur before the onset of disease.

A second reviewer of an earlier version of this paper suggested looking at another population: individuals with chronic gynaecological conditions. Due to the cyclical nature of their symptoms and the high recurrence of disease one could repeatedly assess associations of prodromal content with onset and severity of illness in each cycle. Where a woman is in her cycle can also be objectively verified in relation to potential prodromal dreams and symptom experience.

### Ally with humanities scholars who have studied ancient interpretative practices associated with prodromal dreams

There is now a wealth of material on the ways in which ancient physicians from all over the world used dreams to diagnose and treat illnesses. Beginning in 1945 with the extraordinary work of the Edelsteins (78) and more recently that of Renberg (79) on the Asclepeian traditions of the ancient Hellenic world, it has become clear that not all of those ancient Hellenic dream-medical practices were useless or mere placebo effects. After all, those ancient medical-dream traditions lasted for over a thousand years, involved many tens of thousands patients who sought help at the dozens of dream incubation centers spread all over the ancient Roman empire. There is in addition material records and testimonials of the dreams incubated at these sites and the suggested cures contained in the dreams. There are hundreds of material artifacts in the forms of stone monuments, stelae, iamata and other sources that claim cures from these dreams. In the classical period many cities and towns had sanctuaries dedicated to Asklepios, the main Greek god of healing. There were, however, some Asklepiad sanctuaries which had a prominence greater than others. The temples of healing, the Asklepieia, at the sites of Epidauros, Kos, and Pergamon, achieved status as Panhellenic sanctuaries. The full cycle of Asklepiad ritual was abstinence, ritual bathing, payment of a fee, sacrifice of an animal or food offering, dream incubation, dream interpretation and medical prescription for healing, and thanksgiving when cured by Asklepios. At Corinth patients left behind votive offerings in the form of stone carving or pictures of that part of the body which the god had cured.

### Assess whether dream incubation techniques and networks of dream incubation centers would produce prodromal dreams and improvement in health

Given the ancient testimony and recent scientific work on prodromal dreams I suggest that it is worth considering whether modern dream incubation techniques might help in the study of the effectiveness of prodromal dreaming in moving a person toward better mental and physical health. Techniques for incubation of dreams is undergoing a renaissance of study (see for example, 80–84) Haar Horowitz et al. (85) have actually developed a digital tool to assist in dream incubation. Zhang (86) notes that just as was the case in ancient Mesopotamia and in the Greaco-Roman world, dream incubation rituals had been widely practiced throughout Chinese history. Once again the main aim was to treat illness. In all these ancient cultures there was the use of dream manuals explaining how to properly incubate informative dreams as well as expert dream interpreters assisting in decoding those dreams. Zhang presents the example of the *Secret Book of Praying for Dreams* (SPBD; qi-meng mishu), edited by Shi Shilun (1659–1722). In the Graeco-Roman world virtually all ancient physicians had to become expert at dream interpretation. From Hippocrates to Galen, ancient physicians testify to the usefulness of dreams in their diagnostic and prescriptive practices. However, as mentioned above dream content had to be interpreted soberly and judiciously as dreams often presented riddles or puns and these linguistic metaphors and puns were believed to contain clues to cures. Ancient physicians also asserted that there was a lot of nonsense and superstition surrounding ancient dream interaction beliefs. But sometimes dream content needed no or little interpretation. For Artemidorus of Daldis, a near contemporary of Galen the valuable medical information in dreams was very often presented clearly and without riddles. Often incubated dream content appeared to reflect familiar ancient medical practices, including routine dietary advice, surgical interventions, bathing rituals, and the administration of certain herbal concoctions and drugs. But it is important to note that these medical regimens apparently developed over hundreds of years of experience at these dream incubation sites and across thousands of patients treated at these sites. Much of this cumulative healing knowledge was archived at the Asklepian incubation centers and presumably had to be acquired by the priests at these sites. At most temples dedicated to Asclepius patients purified themselves via abstinence practices and bathing rituals and then summoned by priests to the *abaton* where they would sleep and hope for a dream. Interestingly, if the patient had a dream that involved the “laying on of hands” by the God Asclepius or one of his assistants this was taken as very auspicious for a cure. As the temple priests/dream interpreters came to possess a better understanding of human physiology, and greater cumulative knowledge of what treatments worked or did not work dream content began to reflect updated medical knowledge as it progressed through the centuries.

### Investigate the role of NREM sleep states with regard to potential prodromal content

The role of the NREM sleep states in interoception and prodromal content more generally is not known. My proposed theoretical account of prodromal content focuses only on REM contributions. In addition, some evidence suggests that as we age the NREM sleep states (especially slow wave sleep) declines in power in the EEG record. REM states however do not show as steep a decline as NREM states. The compressed interoceptive information might even become more salient as we age. But again there are not enough studies on this issue to make any definitive statements.

### Explore links between prodromal dreams and other forms of dreaming such as lucid dreaming and hypnogogic dreaming to interrogate interoceptive signals

I have focused on REM-associated dreaming in this essay but there are many other forms of dreams that may be equally potent in indicating emerging illnesses or creative solutions to mental and physical problems. Take for example, dreams that occur when transitioning into or out of stage N1 sleep known as hypnagogic/hypnopompic states (87). In these transitional states the brain tends to forge unusual out of the box semantic connections between otherwise semantically disparate concepts. Lucid dreams (88) represent another opportunity where prodromal information might be present. In lucid dreaming there appears to occur an increased prominence of interoceptive signals. And given that the dreamer is actually conscious he or she may further amplify those signals thus gaining unique information about diseased areas of the body perhaps and so on. In anesthesia-induced dreams Hack et al. (89) showed that these types of dreams may contribute to extremely effective fear extinction. Such cases suggest that anesthetic-induced intra-operative dreaming may be therapeutic in both recent onset and long-standing trauma disorders and have a rapid and durable effect.

## Conclusion

In conclusion, I have presented evidence which suggests that prodromal dreams are possibly worth investigating in the sense that changes in dream content significantly predict emergence of an illness typically soon after those changes in dream content and sometimes years before significant illness occurs. I have also discussed how prodromal dreams are consistent with current theory of the threat simulation functions of both REM sleep brain activation patterns and of dream content. I have discussed links between interoceptive signals and their registration in dream content. I believe that these facts provide an initial rationale that justifies further investigation into prodromal dreams.

## Data Availability

The original contributions presented in the study are included in the article/supplementary material. Further inquiries can be directed to the corresponding author.
